# Diffuse large B cell lymphoma (DLBCL) in patients older than 65 years: analysis of 3 year Real World data of practice patterns and outcomes in England

**DOI:** 10.1038/s41416-021-01525-4

**Published:** 2021-10-05

**Authors:** L. Hounsome, T. A. Eyre, R. Ireland, A. Hodson, R. Walewska, K. Ardeshna, S. Chaganti, P. McKay, A. Davies, C. P. Fox, N. Kalakonda, P. A. Fields

**Affiliations:** 1 Public Health England, London, UK; 2 Department of Haematology, Oxford University Hospitals NHS Foundation Trust, Oxford, UK; 3 Department of Haematology, Kings College London Hospitals, London, UK; 4 Department of Haematology, Ipswich Hospital, Ipswich, UK; 5 Department of Haematology, University Hospitals Dorset, Bournemouth, UK; 6 Department of Haematology, UCLH, London, UK; 7 Department of Haematology, University Hospitals Birmingham, Birmingham, UK; 8 Department of Haematology, Beatson Cancer Centre, Glasgow, UK; 9 Department of Medical Oncology, Southampton General Hospital, Southampton, UK; 10 Department of Haematology, Nottingham University Hospitals NHS Trust, Nottingham, UK; 11 Department of Molecular and Clinical Cancer Medicine, University of Liverpool, Liverpool, UK; 12 Department of Haematology, Guys and St Thomas’ Hospital, London, UK

**Keywords:** B-cell lymphoma, Health policy

## Abstract

**Background:**

We wished to examine treatment and outcome patterns in older diffuse large B-cell lymphoma (DLBCL) patients, with a focus on the effect of route-to-diagnosis to outcome.

**Methods:**

Data were extracted from Public Health England’s National Cancer Registration and Analysis Service between 2013 and 2015 included route-to-diagnosis, disease characteristics and survival for 9186 patients ≥65 years. Systemic Anti-Cancer Therapy data identified front-line regimens, cycles and doses.

**Results:**

Route-to-diagnosis were emergency (34%), NHS urgent cancer pathway (rapid haemato-oncologist review <2 weeks), (29%) and standard GP referral (25%). The most common regimen was R-CHOP (*n* = 4392). 313 patients received R-miniCHOP (7% of R-CHOP). For all patients, 3-year overall survival (OS) for 65–79 years was 57% and for ≥80 years was 32%. Three-year OS for R-CHOP-treated patients diagnosed via emergency presentation was 54% (adjusted hazard ratio (HR) 1.63, *p* < 0.01) and 75% (adjusted HR 0.81, *p* < 0.01) on the NHS urgent cancer pathway (reference HR:1.00: GP referrals). 3-year OS was 54% for both R-miniCHOP and R-CHOP in ≥80 years.

**Conclusions:**

Our comprehensive population analysis is the first to show that the NHS urgent cancer pathway is associated with a superior survival after adjusting for multiple confounders. Equivalent survival for R-CHOP and R-mini-CHOP was demonstrated in those ≥80 years.

## Introduction

Diffuse large B-cell lymphoma (DLBCL) is the most common B cell non-Hodgkin lymphoma (NHL) [1]. Incidence increases with age [2, 3] and presentation is most common in patients over 65 years (https://www.hmrn.org/statistics/incidence). The majority are treated outside of prospective trials with treatment selected by their supervising haemato-oncologists. There is limited population-level data on referral and treatment patterns and patient outcomes. In the UK, the NHS Cancer Plan in the year 2000 (https://webarchive.nationalarchives.gov.uk/20130123203940/, http://www.dh.gov.uk/en/Publicationsandstatistics/Publications/PublicationsPolicyAndGuidance/DH_4010198) [4] announced the NHS urgent cancer referral pathway - the “Two Week Wait” diagnosis pathway initiative the implementation for all suspected cancer patients. This national pathway was developed to expedite an urgent specialist referral from general practice (the GP mandates the patient is seen within 2 weeks by a specialist) for patients whose signs or symptoms were suggestive of cancer. The aim is to enable early diagnostic work up under a specialist, diagnosis and improve outcome. Although clinical trial outcomes are well documented, the outcomes of DLBCL patients treated in a real-world setting across the whole population of England are not described. Specifically, the effect on outcomes by the route to diagnosis, treatment regimen choice, comorbidity and deprivation quintile, have not been described.

DLBCL commonly presents in patients with comorbidities or the very elderly (e.g. ≥80 years). Many of these patients receive R-CHOP (rituximab, cyclophosphamide, doxorubicin, vincristine and prednisolone) or R-CHOP-like therapy based on fitness. To date, no randomised trials comparing cyclophosphamide and doxorubicin dose(s) in elderly DLBCL patients have been performed. Attenuated ‘R-miniCHOP’ is now widely accepted as a standard approach based on the LYSA phase II trial which demonstrated that R-miniCHOP (doxorubicin 25 mg/m^2^, cyclophosphamide 400 mg/m^2^ and vincristine 1 mg capped-dose) provided curative potential in patients ≥80 years [5] with a 2-year PFS and 2-years OS of 47% and 59%, respectively. In light of this study and a subsequent trial of ofatumumab-miniCHOP [6], ‘R-miniCHOP’ has become widely adopted. The question of dose intensity of R-CHOP that addresses disease control and toxicity risk in elderly individuals remains unresolved, with little prospective data to guide decision making. Available retrospective series suggest that in patients ≥80 years, [7–9] there is minimal evidence to support full dose R-CHOP for all patients considered fit for anthracycline-based immunochemotherapy. R-miniCHOP has recently been favoured as the standard arm of the Lymphoma Study Association (LYSA) randomised SENIOR trial in patients ≥80 years [10].

To examine these factors, we sourced data from the National Cancer Registration and Analysis Service (NCRAS), part of Public Health England (PHE). NCRAS maintains the only whole-population comprehensive and mandatory registry of cancer diagnoses in England (https://www.gov.uk/guidance/national-cancer-registration-and-analysis-serviceNCRAS). The registry holds detailed data on each tumour type and is linked to other clinical information such as the Systemic Anti-Cancer Therapy (SACT) dataset. This dataset holds SACT activity from all NHS England providers, sourced from e-prescribing systems [11]. We wished to analyse characteristics and survival outcomes for the DLBCL population in England aged ≥65 years registered in NCRAS (defined by International Classification of Diseases-10 (ICD10) code C83.3). We hypothesised that patients diagnosed and treated following an NHS urgent cancer pathway referral would have improved survival.

## Methods

Data were obtained from PHE NCRAS via a formal application to the organisation’s PHE’s Office for Data Release (ODR). Data were assessed as suitable for release without specific ethical permissions by ODR (ODR assignment no:1617_136) and the NCRAS Caldicott Guardian. All DLBCL diagnoses (ICD10 C83.3) in patients ≥65 years in England in 2013–2015 were identified in NCRAS. At date of extraction, 2013–2015 was the most recent period for which full three-year follow-up of vital status was available. Data were collected for route to diagnosis, extracted on age, sex, ethnicity, stage, comorbidity (modified Charlson Comorbidity index), deprivation index, vital status, and cause of death. The deprivation measure was the Income domain of English Indices of Deprivation 2010 (see supplement for further details) (https://www.gov.uk/government/statistics/english-indices-of-deprivation-2010). The route to diagnosis is a derived measure of the way in which a patient arrived at their cancer diagnosis, based on a combination of several datasets [12]. Data on frontline chemotherapy was obtained from SACT and included regimens, cycle dates, treatment intent, and regimen modifications (e.g. R-miniCHOP). All relevant comorbid conditions were assigned a weight from 1 to 6, based on specific diagnosis codes found in hospital admissions in a 3–27 month period prior to DLBCL diagnosis. These weights were then summated to produce an abbreviated comorbidity index. Fig. S1 provides a summary of the methodology and timelines of accessing and linking relevant data.

Data were linked and analysed using Stata 15 (StataCorp. 2017. College Station, TX: StataCorp LLC). Kaplan–Meier survival curves were generated using STS command. Overall survival (OS) was defined from DLBCL diagnosis until time of death from any cause and differences in OS were determined by log-rank tests. Lymphoma-specific survival (LSS) was defined from DLBCL diagnosis until the time of death from DLBCL, with censoring occurring at the cause of death for other unrelated causes other than DLBCL. To account for the impact of multiple variables on survival, a Cox-regression model was implemented. Differences in proportions for descriptive statistics were tested with a 2-sided Z-test. Data were interrogated to explore potential associations between route to diagnosis and baseline characteristics with OS and LSS. We also evaluated R-CHOP intended dose intensity (IDI) across the population—with a particular focus on very elderly (≥80 years). Patients were analysed according to categories 65–79 and ≥80 years as dose intensity decisions typically vary according to those age cut-offs. IDI was determined by strict first cycle total dosage analysis of individual drug components (defined for R-miniCHOP: vincristine 1 mg, doxorubicin ≤55 mg, and cyclophosphamide ≤880 mg). These thresholds were chosen to include all patients receiving R-miniCHOP up to a maximum body surface area of 2.2 m^2^ based on the standard R-miniCHOP doses (400 mg/m^2^ cyclophosphamide, 25 mg/m^2^ doxorubicin). R-CHOP was defined as either cyclophosphamide or doxorubicin doses greater than R-miniCHOP defined thresholds. The study was performed in accordance with the Declaration of Helsinki. All authors had full access to the data and the corresponding author had final responsibility for the decision to submit the manuscript for publication.

## Results

A total of 9186 DLBCL patients ≥65 years across a 3-year period (01/01/2013–31/12/2015) were identified in the NCRAS database. Table 1 describes key baseline characteristics. Median follow-up of the whole cohort was 2.1 years. The cohort was largely aged 65–79 years (*n* = 6203, 68%). Gender distribution was broadly equal. Less deprived population quintiles (quintiles 1–2) had numerically more cases. Men were more likely to be younger (55% of those 65–79 years vs 49% of those ≥80 years; *p* < 0.001) and with lower levels of deprivation (56% in quintile 1 vs 51% in quintile 5; *p* = 0.008). Across all patients, 4536 presented with stage III–IV (61% of available data) and 2918 presented with early-stage DLBCL (39%). The proportion diagnosed with advanced stage decreased with increasing age (63% in 65–79 years vs 56% in ≥80 years; *p* < 0.001). Characteristics according to year were similar (data not shown)

**Table 1:** Baseline characteristics according to age.

Baseline characteristics		Age 65–79 (*n* = 6203)	Age ≥ 80 (*n* = 2983)	All ages (*n* = 9186)
Sex	Male	3414 (55%)	1455 (49%)	4869 (53%)
	Female	2789 (45%)	1528 (51%)	4317 (47%)
Stage	Early	1895 (37%)	1023 (44%)	2918 (39%)
	Advanced	3229 (63%)	1307 (56%)	4536 (61%)
	Missing	1079	653	1732
Performance status (ECOG)	0	912 (42%)	211 (29%)	1123 (39%)
	1	753 (35%)	276 (38%)	1029 (35%)
	2	496 (23%)	249 (34%)	745 (26%)
	Missing	4042	2247	6289
Number of co morbid conditions	0	4468 (72%)	1955 (66%)	6423 (70%)
	1	780 (13%)	449 (15%)	1229 (13%)
	2	563 (9%)	283 (9%)	846 (9%)
	≥3	392 (6%)	296 (10%)	688 (7%)
Deprivation quintile	1—Least deprived	1380 (23%)	686 (24%)	2066 (23%)
	2	1427 (24%)	694 (24%)	2121 (24%)
	3	1300 (21%)	606 (21%)	1906 (21%)
	4	1141 (19%)	563 (19%)	1704 (19%)
	5—Most deprived	805 (13%)	361 (12%)	1166 (13%)
	Missing	150	73	223
Route to diagnosis	Emergency	2051 (34%)	1108 (38%)	3159 (35%)
	GP referral	1573 (26%)	676 (23%)	2249 (25%)
	Inpatient Elective	82 (1%)	31 (1%)	113 (1%)
	Other outpatient	606 (10%)	243 (8%)	849 (9%)
	NHS urgent cancer referral pathway	1752 (29%)	883 (30%)	2635 (29%)
	Missing	138	42	180

### Route to diagnosis

Across all patients, the most common route to diagnosis was emergency presentation (35%). A substantial proportion also presented via the NHS urgent cancer pathway referral (29%) and a standard elective GP referral (timed ‘two week wait pathway’ not mandated) (25%). Nine percent of patients were referred from other outpatient services. Emergency presentation was more common in advanced stage disease; of those presenting via emergency route, 69% had stage III–IV disease. In contrast, the most common route to diagnosis for early-stage disease was via the NHS urgent cancer referral pathway. There were no differences in emergency presentation proportion by sex, however, emergency presentations were more common in older patients (37% in ≥80 years vs 33% in 65–79 years; *p* < 0.001). Baseline characteristics were available for 4392 R-CHOP-treated patients and are described in Table S1. Again, R-CHOP patients presenting as an emergency were significantly more likely to present with advanced stage disease compared to GP referrals or NHS urgent cancer referral pathway. Other demographics were relatively well matched across the diagnostic routes.

### Treatment regimen

Of patients with a recorded regimen, the most common was R-CHOP (*n* = 4392, 48%), followed by R-CVP (rituximab, cyclophosphamide, vincristine, prednisolone) (6%), R-CEOP (rituximab, cyclophosphamide, etoposide vincristine, prednisolone) (2%), R-PMITCEBO (1%), R-EPOCH (rituximab, etoposide, prednisolone vincristine, cyclophosphamide, doxorubicin) (0.5%) and R-GCVP (rituximab, gemcitabine, cyclophosphamide, vincristine, prednisolone) (0.5%). Only 2% received first-line therapy within an interventional clinical study protocol. Approximately 40% had either no SACT regimen recorded or had another treatment outside of those described. Within that group, 1226 patients (13.3%) received ‘chemotherapy of unspecified type’ and 599 patients (6.5%) received radiotherapy alone and 814 patients (8.9%) were known to receive palliative care support alone. Overall, R-CHOP represents 80.5% (4392/5453) of all DLBCL cases with a known frontline regimen. Trials listed in SACT were INCA [13], PHOENIX [14], and REMODL-B [15].

### Survival outcomes

**Fig. 1:**
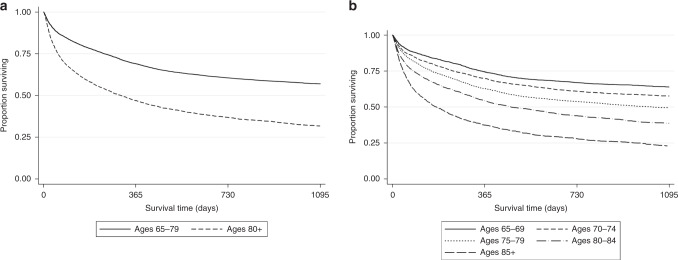
Overall survival all DLBCL patients by age. **a** age 65–79 vs ≥80 years, **b** ages in 5-year subgroups.

**Fig. 2:**
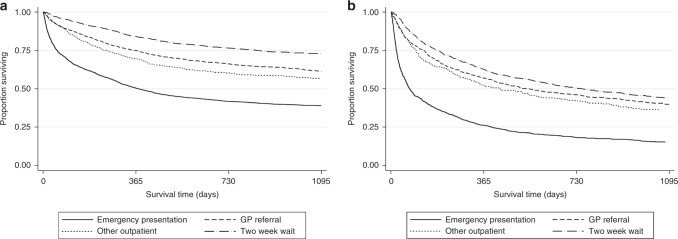
Overall survival all patients DLBCL by route to diagnosis by age. **a** age 65–79 years (**b**) age ≥80 years.

OS outcomes for all 9186 patients are shown in Fig. 1. 4819 (52%) had died; with 1047 (22%) of these recorded as due to DLBCL. Three-year OS was 49% (95% CI 48–50%). Three-year OS for those aged 65–79 years was higher at 57% (95% CI 56–58%) than for those aged ≥80 years at 32% (95% CI 30–33%) (*p* < 0.001) (Fig. 1a). OS progressively worsened with advancing age (Fig. 1b). Three-year OS for 65–69, 70–74, 75–79, 80–84 and ≥85 years were: 64%, 58%, 50%, 39% and 23%, respectively (*p* < 0.001 for trend). Three-year OS was 65% for patients receiving the regimens listed, 44% for all other regimens and 26% when no treatment was recorded by SACT (*p* < 0.001). 3-year OS for the NHS urgent cancer referral pathway was 74% (95% CI 72–76%), for GP referrals 68% (95% CI 66–71%), for other outpatients 68% (95% CI 63–73%) and for emergency presentation 53% (95% CI 50–56%). Survival according to stage and route of presentation (Fig. 2S) shows that patients who have both early or late-stage DLBCL presenting as an emergency have an inferior OS compared with any other subgroup.

### Univariable (UVA) and multivariable survival analysis (MVA)

#### Route of presentation

Table 2 presents the OS univariable (UVA) and multivariable (MVA) Cox-regression analysis for 4392 R-CHOP-treated patients. The 1635 patients who presented via the NHS urgent cancer referral pathway had a 22% lower risk of death on UVA compared to those presenting via routine elective GP referral (*p* < 0.01). This significant effect was retained on MVA (hazard ratio (HR) 0.81, 95% CI 0.71–0.93, *p* < 0.01). In contrast, 1160 patients presenting via the emergency route displayed a 63% increased risk of death on MVA (HR 1.63, 95% CI 1.43–1.86, *p* < 0.01).

**Table 2:** Cox-regression analysis for overall survival for all R-CHOP treated patients.

			Univariate analysis				Adjusted multivariable analysis			
Variable		Number of patients	HR	HR (CI)		*p*-value	HR	HR (CI)		*p*-value
Route to diagnosis	GP referral	1110	Ref	–	–	–	Ref	–	–	–
	Emergency presentation	**1160**	**1.68**	**1.48**	**1.92**	**<0.01**	**1.63**	**1.43**	**1.86**	**<0.01**
	Inpatient elective	51	0.78	0.46	1.32	0.36	0.83	0.48	1.42	0.50
	Other outpatient	374	0.97	0.80	1.19	0.81	0.96	0.78	1.18	0.70
	NHS urgent cancer referral pathway	**1635**	**0.78**	**0.68**	**0.89**	**<0.01**	**0.81**	**0.71**	**0.93**	**<0.01**
	Unknown	**61**	**0.45**	**0.24**	**0.84**	**0.01**	**0.52**	**0.28**	**0.98**	**0.04**
Age	Five-year increment	**65**–**69 (*n*** = 1265) **70**–**74 (*n*** = 1143) **75**–**79 (*n*** = 1080) **80**–**84 (*n*** = 670) ≥**85 (*n*** = 234)	**1.05**	**1.04**	**1.06**	**<0.01**	**1.05**	**1.04**	**1.06**	**<0.01**
Sex	Male	2311	Ref	–	–	–	Ref	–	–	–
	Female	**2081**	**0.83**	**0.75**	**0.92**	**<0.01**	**0.83**	**0.75**	**0.91**	**<0.01**
Year (2013–2015)	Yearly increment	2013 (*n* = 1338) 2014 (*n* = 1452) 2015 (*n* = 1602)	0.98	0.92	1.05	0.63	0.97	0.91	1.03	0.29
Deprivation quintile	Increment of quintile	1 (least deprived) (*n* = 1014) 2 (n = 1020) 3 (*n* = 921) 4 (*n* = 802) 5 (most deprived) (*n* = 530) Unknown *n* = 105	**1.04**	**1.00**	**1.07**	**0.05**	1.03	0.99	1.07	0.09
Stage	Early	1497	Ref	–	–	–	Ref	–	–	–
	Advanced	**2342**	**1.73**	**1.53**	**1.95**	**<0.01**	**1.73**	**1.53**	**1.95**	**<0.01**
	Unknown	**553**	**1.66**	**1.41**	**1.96**	**<0.01**	**1.61**	**1.37**	**1.92**	**<0.01**
Number of co morbid conditions	Increment of index	0 (*n* = 3252) 1 (*n* = 526) 2 (*n* = 397) 3 (*n* = 123) 4 (*n* = 51) 5 (*n* = 27) 6 (*n* = 9) 7 (*n* = 4) 8 (*n* = 2) 9 (*n* = 1)	**1.18**	**1.13**	**1.23**	**<0.01**	**1.15**	**1.10**	**1.20**	**<0.01**

*HR* hazard ratio, *CI* confidence intervals at 95%.

Statistically significant *p*-values are in bold.

These findings were similar across the whole population. Figure 2 shows outcomes according to age and route of presentation in all patients. Three-year OS was superior for patients referred by the NHS urgent cancer referral pathway (65–79 years: 73%, ≥80 years: 44%) and inferior by the emergency route (65–79 years: 39%, ≥80 years: 15%) (Fig. 2a, b)

#### Other baseline factors

According to MVA, each 5-year age increment led to a 5% increased risk of death. Female patients had a 17% reduced risk of death compared to men. In an adjusted model, patients with late stage disease were at a 73% increased risk of death when compared to early stage. Patients with an higher comorbidity burden were also at increased risk of death, with each increment of comorbidity increasing risk by 15%. Overall, patients with late stage disease, increased deprivation, male gender, and increased age (by 5-year increment) all had an independently inferior survival. The results from an adjusted Cox-regression analysis for LSS were similar. The notable difference was that comorbidity index became insignificant as a predictor of LSS (HR 0.97, 95% 0.87–1.08, *p* = 0.60). Importantly, the NHS urgent cancer referral pathway retained its association with an improved LSS (HR 0.71, 95% CI 0.53–0.95, *p* = 0.02), whilst patients with an emergency presentation had a significantly worse LSS by MVA (HR 1.95, 95% CI 1.49-2.54, p < 0.01) (Table S2).

### Outcomes according to treatment regimen

#### R-CHOP

**Fig. 3:**
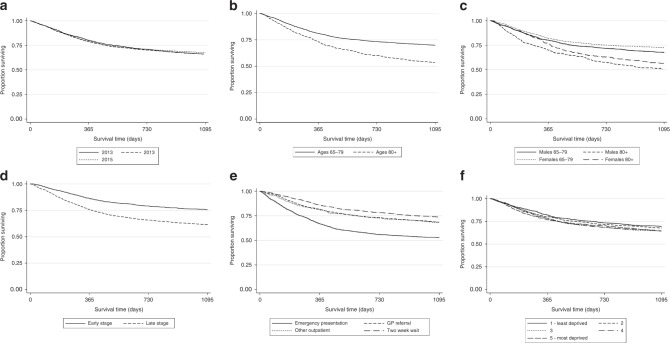
Overall survival of all R-CHOP treated patients by. **a** year of diagnosis (**b**) by age 65–79, ≥80 years (**c**) age and sex (**d**) stage at diagnosis (**e**) route to diagnosis (**f**) deprivation.

4392 patients received R-CHOP at either full or ‘attenuated’ dosing including 4079 patients receiving R-CHOP at dosing greater than R-miniCHOP. Three-year OS was 68% (95% CI 66–69%) and did not differ across years analysed (Fig. 3a) (*p* = 0.82). When analysed by age, 3-year OS was 70% (95% CI 69–72%) for 65–79 years and 54% (95% CI 49–57%) for ≥80 years (Fig. 3b) (*p* < 0.001). When analysed by age and sex, the highest three-year OS was observed in females aged 65–79 years (3-year OS 73%) and lowest OS for men ≥80 years (3-year OS 51%) (Fig. 3c) (*p* < 0.001). For patients with advanced stage disease, three-year OS was 62% (95% CI 60–64%) and for early-stage disease 77% (95% CI 75–79%) (Fig. 3d) (*p* < 0.001). A significantly inferior OS was seen in patients with emergency presentation: 3-year OS 54% (95% CI 51–57%), and the highest observed OS of 75% (95% CI 72–77%) for the NHS urgent cancer referral pathway group (Fig. 3e) (*p* < 0.001). Diagnosis to treatment interval was assessed in 3532 R-CHOP treated patients where data were available. DTI of 0–29 days was strongly associated with statistically shorter OS compared to DTI of 30+ days (*p* < 0.001) (Fig. 3Sa). DTI was shorter in patients with emergency presentation (0–14 days 35.4%, 15–29 days 34.8%, chi-squared *p* < 0.001) compared to GP referrals. NHS urgent cancer referral pathway DTI were statistically longer than GP referrals (Urgent NHS referral: 0–14 days 9.7%, 15–29 days 32.5%, GP referral: 0–14 days 13.5%, 15–29 days 28.7%, chi-squared *p* < 0.001) (Fig. 3Sb).

There was a statistically significantly lower OS in more deprived groups (*p* = 0.01). The effect size, however, was small: 3-year OS in least deprived was 69% compared to 65% in most deprived (Fig. 3f).

#### R-miniCHOP

**Fig. 4:**
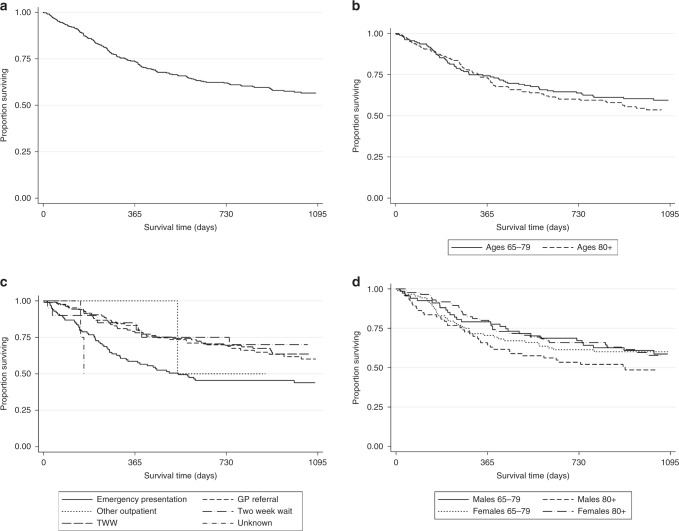
Overall survival R-miniCHOP treated patients by. **a** all patients (**b**) by age 65–79, ≥80 years (**c**) route to diagnosis (**d**) age and sex.

Of the R-CHOP-treated patients, 313 patients (7%)) received R-miniCHOP. Three-year OS for the R-miniCHOP group was 57% (95% CI 51%-62%) (Fig. 4a). Patients 65-79 years who received R-miniCHOP had a numerically higher comorbidity burden compared to those receiving standard R-CHOP (comorbidity index ≥1: 25% R-CHOP versus 35% for R-miniCHOP). When stratified by age (Fig. 4b), the 65–79 years group demonstrated a 3-year OS of 59% (95% CI 51–67%) and ≥80 years group 54% (95% CI 45–61%). Patients presenting via the emergency route had an inferior outcome as did males ≥80 years (Fig. 4c, d). Two-year OS for patients ≥80 years was 60% (95% CI 52–67%).

#### R-CHOP versus R-miniCHOP in patients ≥ 80 years

**Fig. 5:**
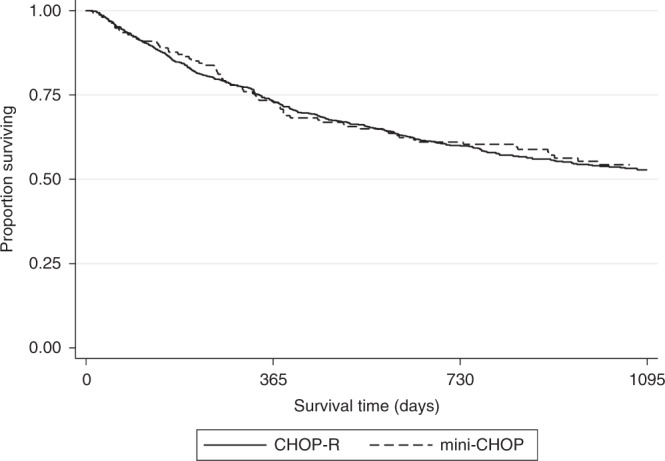
OS for patients aged ≥80 years, treated with either R-CHOP or R-mini-CHOP.

Table 3 describes the characteristics of patients ≥80 years divided by dose intensity. Proportionally more patients ≥85 years received R-miniCHOP, but otherwise the cohorts were strikingly well balanced according to stage, comorbidity, gender and deprivation index. Table S3 describes cycles received in patients ≥80 years by dose intensity. Despite equivalent staging, patients receiving R-miniCHOP had a numerically higher proportion of patients who received 6–8 cycles compared to R-CHOP (54 vs 43%) and less patients who received only 1–3 cycles (29 vs 38%) which may reflect disease stage. Figure 5 shows the OS of patients ≥80 years treated with R-CHOP (*n* = 746) or R-miniCHOP (*n* = 158). OS curves completely overlap, with the 3-year OS for both groups equalling 54%. There was no difference in LSS for the 2 groups either (Fig. 4S). When 73 patients ≥80 years receiving full dose R-CHOP were compared to the 202 patients ≥80 years receiving ≥1 chemotherapeutic at full dose (but not all chemotherapeutics), there was no statistically significant difference in OS ((full dose 3-year OS 60.1% (CI 48.9–74.0%) vs. at least 1 chemotherapeutic at full dose 3-year OS 53.4% (CI 46.8–60.9%)). Regimen intensity (R-CHOP or R-miniCHOP) was included with an adjusted MVA of patients exclusively ≥80 years (Table S4). Whilst late-stage disease (HR 1.31, *p* = 0.01), emergency presentation (HR 1.82 (*p* < 0.01) and age (HR 1.06 per age bracket, *p* < 0.01) were adverse prognostic features and female sex (HR 0.82, *p* = 0.04) a good prognostic factor, the choice of R-CHOP or R-miniCHOP had no influence over OS (R-CHOP reference, HR 0.95, 95% CI 0.73–1.22, *p* = 0.68).

**Table 3:** Baseline characteristics in patients 80 years and over according to dose intensity of R-CHOP.

Baseline characteristic		R-CHOP		Mini-R-CHOP	
		Number of patients		Number of patients	
Age	80–84 years	564	76%	106	67%
	≥85 years	182	24%	52	33%
Sex	Male	392	53%	73	46%
	Female	354	47%	85	54%
Deprivation quintile	1—least deprived	185	25%	41	26%
	2	183	25%	36	23%
	3	152	21%	30	19%
	4	127	17%	22	14%
	5—most deprived	83	11%	20	13%
	Unknown	16	2%	9	6%
Stage at diagnosis	Early	311	42%	68	43%
	Late	335	45%	69	44%
	Unknown	100	13%	21	13%
Comorbidity index	0	583	72%	110	70%
	1	89	12%	20	13%
	2	71	10%	17	11%
	≥3	48	6%	11	7%

#### Non-anthracycline-containing regimens

Three-year OS was 52% (95% CI 44–59%) for non-anthracycline-containing regimens (R-CEOP (*n* = 166), R-GCVP (*n* = 37), total *n* = 203) (Fig. 5Sa), with numerically higher OS in those 65–79 years (54%; 95% CI 44–63%) compared to ≥80 years (49%; 95% CI 38–60%) (*p* = 0.42) (Fig. 5Sb). In this small subgroup 3-year OS after routine GP referral was 64% (95% CI 48–77%), NHS urgent cancer referral was 55% (95% CI 42–66%), and OS after emergency presentation was 39% (95% CI 26–51%) (Fig. 5Sc). The 3-year OS for the 553 R-CVP-treated patients was 43% (95% CI 38–47%) and for 166 R-CEOP treated patients was 54% (46–52%) (Fig. 6Sa, b). When the 325 patients receiving R-CVP only with no hybrid regimens included were analysed specifically, the 3-year OS was equivalent (3-year OS 44.1% (95% CI 38.9–50.0%).

## Discussion

We present the analysis of a large real-world DLBCL dataset of an elderly (≥65 years) DLBCL population diagnosed over a three-year consecutive period (2013–2015). We report four key findings that we believe are generalisable to elderly DLBCL patients. Firstly, to our knowledge, the DLBCL survival outcomes presented are the first to describe in an adjusted analysis, that timed diagnostic NHS urgent cancer referral pathway (rapid early haemato-oncology access within 14-days) produced the most superior overall and lymphoma-specific survival. Secondly, patients presenting as an emergency at diagnosis have an inferior overall and lymphoma-specific survival on MVA when compared to standard non-2 week wait GP referrals. Thirdly, patients ≥80 years have equivalent OS whether they receive R-CHOP or R-miniCHOP. Fourthly, elderly males have the worst OS of all groups studied.

Since the NHS timed urgent cancer referral pathway was implemented in the year 2000, analyses demonstrating its effectiveness in improving survival across solid cancers have been mixed. Some solid cancer series have suggested little benefit [16, 17] although a large meta-analysis demonstrated worse outcomes where diagnosis and treatment are delayed [18]. A small study in DLBCL did not show that time-to-diagnosis influenced OS [19]. In the first comprehensive nationwide analysis of its kind in DLBCL, we report the most superior outcomes were recorded for 1635 R-CHOP-treated patients presenting on a timed NHS urgent cancer referral pathway via “the Two Week Wait” where patients’ investigations and diagnosis are expedited as quickly as possible. The pathway mandates that patients see a specialist within 14 days and enter treatment within 62 days of initial primary care referral. Our data suggest that this pathway reduces risk of mortality in R-CHOP treated patients compared with standard GP referrals. These findings in part validate this strategy in enhancing DLBCL patient outcomes. DLBCL is a proliferative and aggressive disease where rapid diagnostic pathways make clinical sense. Our findings also suggest that, in order to improve future outcomes, reducing the proportion of patients presenting as an emergency is desirable. Emergency presentation had the lowest recorded survival and was also the most commonly observed diagnostic route (34%). This may in part be due to patients not presenting with early detectable signs or symptoms, or a failure to recognise signs and symptoms of lymphoma although this simply may represent aggressive disease biology with adverse clinical features in some patients. We cannot be certain that better education will improve outcomes for all patients, particularly those who present with a clinical presentation requiring emergency care. However, to improve early recognition, national patient associations (e.g. Lymphoma Action, Blood Cancer UK) have developed tool kits designed to aid primary care practitioners’ knowledge in recognition of the early presenting signs and symptoms of the disease, which may be subtle and not always immediately clinically apparent. Whether the NHS urgent cancer referral pathway results in improved OS in other haematological cancers remains an open, unexplored question.

There is a further drive by NHS England to diagnose cancer as early as possible [20] (2019 NHS plan), by raising symptom awareness, and implementing faster diagnosis standards through the roll out of rapid diagnostic centres. Moreover, recent UK population-based analysis have also described the potential impact of COVID19-related disruption to the NHS urgent cancer pathway and the potential negative impact on long term OS this may have [21, 22].

Recent data [23, 24] has clearly demonstrated that diagnosis-to-treatment interval (DTI) in DLBCL and other aggressive NHL has an inverse effect on event-free survival. Our data has validated this in a population-based setting and also suggests that shorter DTI is more prevalent in patients presenting as an emergency as might be expected. The short time-to-diagnosis that the NHS urgent cancer pathway strategy enables provides decision-making control for the patient and physician regarding pace of DTI.

In our series, elderly males demonstrated the poorest OS (3-year OS 51%). This finding mirrors published clinical trial data [25–27] and non-trial data [28] where confirmatory large series of studies demonstrated that elderly men achieve inferior outcomes when compared to their age-matched female counterparts. Recently, the HOVON-84 randomised clinical trial demonstrated that increased intensification of rituximab dosing early in treatment alongside CHOP-14 did not improve outcomes in DLBCL patients between 18 and 80 years compared to those receiving R-CHOP-14 [29]. Pharmacokinetic data has demonstrated enhanced clearance of the anti-CD20 in elderly males [25]. Whether enhancing rituximab dosage in trials specifically powered to assess in elderly patients remains unanswered.

Our data also demonstrate inferior outcomes for patients who present with pre-existing medical co-morbidities; in line with previous large population studies of DLBCL[30] in other countries. A similar outcome is not observed for lymphoma-specific survival; a possible consequence of competing mortalities resulting from significant co-morbidities.

The most common regimen choice was R-CHOP (48% of all cases, 80.5% of known front-line regimen) with recorded outcomes consistent with population studies from the US [31] and Sweden [32]. Approaches to enhance these outcomes have proven difficult in the front-line setting. Recent studies have attempted to improve outcomes by the addition of novel agents to the R-CHOP backbone. For example, the addition of ibrutinib to R-CHOP enhanced efficacy in patients <60 years but was associated with enhanced toxicity in those >60 years [14].

Many elderly patients are not suitable for full dose intensity R-CHOP. Options exist for these patients are undoubtedly influenced by performance status, and the presence of pre-existing comorbid conditions. Such patients often receive dose attenuated regimens such as R-miniCHOP [5]. Our series contained 313 patients treated with R-miniCHOP and reported an almost identical two-year OS of 60% in those ≥80 years as reported in the LYSA trial. Three-year OS of 54% confirmed its efficacy across a broader real-world population. We also report 155 patients in the 65–79-year group unable to receive full dose intensity R-CHOP but who received R-miniCHOP. Three-year OS of 59% suggests younger patients may derive considerable benefit from dose attenuated regimens when they are otherwise clinically unsuitable to receive standard R-CHOP due to frailty or dose-limiting comorbidities. We also report a similar 3-year OS of 54% in a large R-CEOP cohort and recognise this option is a valid alternative approach in these patients who may otherwise be considered for R-miniCHOP due to frailty or cardiac comorbidity.

A further area of uncertainty is the importance of dose intensity of R-CHOP in those ≥80 years. There is a theoretical risk of inferiority of dose attenuation when compared to full dose R-CHOP as several small studies have documented that retaining dose intensity improves outcomes in younger patients [33–36]. However, in the largest series analysed to date, we demonstrate that survival outcomes for R-CHOP (*n* = 746) and R-mini-CHOP (*n* = 158) treated patients ≥80 years were superimposable, confirming the equivalence of R-mini-CHOP to higher dose intensity R-CHOP in this subgroup. We do recognise that patients included in the R-CHOP group may have received some degree of dose attenuation between full and R-CHOP doses and the data should therefore be interpreted in light of this.

Our study has several limitations. It would have been desirable to have more complete data including full IPI components and other factors known to be associated with an inferior outcome in DLBCL. It remains possible that unmeasured confounders may have influenced results. For example, patients presenting as an emergency could have a higher proportion of extranodal disease sites and more bulky disease; factors known to be associated with inferior outcome. Based on our results, we cannot state definitively if the NHS urgent cancer pathway strategy helps patients with these additional factors or whether inferior disease biology specifically is the primary driver of this presentation in these patients. We do however note that early-stage patients presenting as an emergency have an inferior OS than all other groups other than late-stage emergency presentations, which suggests that multiple extranodal nodal sites or overall disease burden may not be the only driver to worse outcomes seen.

It would also be desirable to present a more granular analysis of relative dose intensity. Despite this, large recent studies show that relative dose intensity correlates very closely with intended dose intensity, with intended dose intensity the significant driving factor associated with survival [9]. We also recognise the lack of specific comorbidity classifications e.g. cardiac and lack of prognostic biological data (e.g. cell of origin, MYC/BCL2 status). These factors may have minimised the effect of unmeasured potential confounding variables.

It is possible that the lymphoma-specific survival rates observed in this cohort are higher than those previously observed in equivalent national studies [7] due to under recording of DLBCL as a specific cause of death. Care should be taken when comparing lymphoma-specific survival within our cohort to other studies outside the UK as procedures for recording causes of death on the death certificate, and the analytic methods used could vary. Nevertheless, outcomes within the MVA are consistent with the OS analysis and are clinically relevant.

We also recognise that the overall recording of comorbidities is relatively low for patients within ages studied. This may be attributable to low numbers of comorbid illnesses being recorded during inpatient episodes and the inability to record co-morbid illnesses in all patients treated in a non-inpatient setting. We recognise that national, systematic comorbidity data collection methods are needed to ensure all comorbidities are recorded across the hospital episodes and primary care. Further efforts to improve the availability of primary care data to capture such comorbidity data will provide more information going forward to optimise future analyses.

Finally, we note that 40% of patients in the total dataset had no treatment information recorded or had another treatment outside those analysed. Overall, these patients had an inferior survival to those patients with treatment detailed in the manuscript. This, therefore, represents a large, unexplored cohort within the national registry. We recognise the importance of high-quality palliative care and palliative therapies in this setting but acknowledge that a detailed analysis of these patients in outside the scope of this manuscript.

## Conclusion

We have demonstrated that patients who are referred via the timed ‘2-week wait’ NHS urgent cancer referral pathway where initial investigations are performed within 14 days achieved the best documented survival outcomes. We also confirm the efficacy of the R-miniCHOP regimen based on intended dose intensity data in patients ≥80 years and its probable equivalence to R-CHOP in a real-world setting. In our patient cohort, we have observed that elderly patients over 80 years have significantly inferior outcomes—in particular elderly males. This necessitates further research into causation of this gender discrepancy and the use of novel non-chemotherapeutic targeted agents to improve outcomes in elderly and frail DLBCL patients.

## Supplementary information

Supplementary Material

## Data Availability

For data sharing and availability, please contact the corresponding author, Prof. Paul Fields.
