# Supplementary material for: Diffuse large B cell lymphoma (DLBCL) in patients older than 65 years: analysis of 3 year Real World data of practice patterns and outcomes in England
Source: Br J Cancer. 2021 Oct 5;126(1):134–43. doi: 10.1038/s41416-021-01525-4 (PMC8727618; doi:10.1038/s41416-021-01525-4)
Supplement: Supplementary file 1 — Supplementary Material [file 41416_2021_1525_MOESM1_ESM.docx]

**Supplementary Material**

**The concept of multiple deprivation: The English Indices of Deprivation 2010**

Each domain represents a specific form of deprivation experienced by people and each can be measured individually using a number of indicators. Seven distinct domains have been identified in the English Indices of Deprivation: Income Deprivation, Employment Deprivation, Health Deprivation and Disability, Education Skills and Training Deprivation, Barriers to Housing and Services, Living Environment Deprivation, and Crime. Individual domains can be used in isolation as measures of each specific form of deprivation. They can also be combined, using appropriate weights, into a single overall Index of Multiple Deprivation which can be used to rank every small area in England according to the deprivation experienced by the people living there. Along with two supplementary indices, Income Deprivation Affecting Children Index and Income Deprivation Affecting Older People Index, they form the English Indices of Deprivation. These statistics allow the most, and least, deprived areas of the country to be identified as well as providing information about the issues faced by people living in different parts of the country.

Relevant Information taken from: https://assets.publishing.service.gov.uk/government/uploads/system/uploads/attachment_data/file/6871/1871208.pdf

**Supplementary Figure 1:** Flow diagram Data extraction and analysis

**Supplementary Figure 2:** Overall survival according to both stage and route of DLBCL presentation

**Supplementary Figure 3A:** OS by Diagnosis to treatment interval (DTI)

**Supplementary Figure 3B:** Diagnosis to treatment by route of referral

**Supplementary Figure 4:** Lymphoma-Specific Survival for R-CHOP and R-miniCHOP in patients ≥80 years

**Supplementary Figure 5:** Overall survival non-anthracycline treated patients by: a) all patients b) by age 65-79, ≥80 years c) Route to Diagnosis

**Supplementary Figure 6:** Overall survival for all R-CVP and R-CEOP treated patients

**Supplementary Table 1: Patient characteristics according to route of presentation for 4,392 R-CHOP treated patients**

| **Baseline Characteristics** | **Route to Diagnosis** | | | | | |  |
| --- | --- | --- | --- | --- | --- | --- | --- |
| **Variable** | **Emergency** | **GP referral** | **Inpatient** | **Other outpatient** | **NHS urgent cancer referral pathway** | **Unknown** | **Total number** |
| **65-79** | 80% | 81% | 82% | 82% | 76% | 90% | 3,488 |
| **≥80** | 20% | 19% | 18% | 18% | 24% | 10% | 904 |
|  |  |  |  |  |  |  |  |
| **Male** | 54% | 51% | 65% | 57% | 52% | 48% | 2,311 |
| **Female** | 46% | 49% | 35% | 43% | 48% | 52% | 2,081 |
|  |  |  |  |  |  |  |  |
| **1 - least deprived** | 22% | 25% | 22% | 27% | 22% | 38% | 1,014 |
| **2** | 21% | 23% | 29% | 24% | 25% | 25% | 1,020 |
| **3** | 22% | 20% | 22% | 18% | 22% | 21% | 921 |
| **4** | 20% | 18% | 10% | 16% | 18% | 7% | 802 |
| **5 - most deprived** | 13% | 12% | 16% | 13% | 11% | 8% | 530 |
| **Missing** | 2% | 3% | 2% | 2% | 2% | 2% | 105 |
|  |  |  |  |  |  |  |  |
| **Early** | 24% | 34% | 37% | 33% | 41% | 31% | 1,497 |
| **Late** | 63% | 652% | 53% | 51% | 48% | 49% | 2,342 |
| **Missing** | 13% | 14% | 10% | 15% | 11% | 20% | 553 |
| **Comorbidity index** |  |  |  |  |  |  |  |
| **0** | 73% | 69% | 75% | 66% | 80% | 80% | 3,252 |
| **1** | 13% | 13% | 16% | 11% | 11% | 5% | 526 |
| **2** | 9% | 12% | 10% | 14% | 6% | 11% | 397 |
| **≥3** | 6% | 6% | 0% | 8% | 3% | 3% | 217 |

**Supplementary Table 2: Adjusted Cox-regression for Lymphoma-Specific Survival**

| **Baseline characteristics** | | ***HR** | **HR (95% CI)** | | **p-value** |
| --- | --- | --- | --- | --- | --- |
| **Route to diagnosis** | GP referral | Ref | - | - | - |
|  | Emergency presentation | **1.95** | **1.49** | **2.54** | **<0.01** |
|  | Inpatient elective | 1.00 | 0.36 | 2.70 | 0.99 |
|  | Other outpatient | 0.72 | 0.44 | 1.17 | 0.18 |
|  | NHS urgent cancer referral pathway | **0.71** | **0.53** | **0.95** | **0.02** |
|  | Unknown | - | - | - | - |
|  |  |  |  |  |  |
| **Age** | Five-year increment | **1.05** | **1.04** | **1.07** | **<0.01** |
|  |  |  |  |  |  |
| **Sex** | Male | Ref | - | - | - |
|  | Female | **0.76** | **0.62** | **0.94** | **0.01** |
|  |  |  |  |  |  |
| **Year (2013-2015)** | Yearly increment | **1.16** | **1.01** | **1.32** | **0.03** |
|  |  |  |  |  |  |
| **Deprivation quintile** | Increment of quintile | **1.10** | **1.02** | **1.18** | **0.01** |
|  |  |  |  |  |  |
| **Stage** | Early | Ref | - | - | - |
|  | Late | **2.05** | **1.58** | **2.66** | **<0.01** |
|  | Unknown | **1.60** | **1.09** | **2.33** | **0.02** |
|  |  |  |  |  |  |
| **Comorbidity index** | Increment of index | 0.97 | 0.87 | 1.08 | 0.60 |

****HR = hazard ratio , CI = confidence intervals at 95%***

**Supplementary Table 3: cycles of R-CHOP delivered in patients ≥80 years receiving R-CHOP**

| **Cycles of RCHOP delivered** | **R-CHOP (n=746)** | **R-miniCHOP (n=158)** |
| --- | --- | --- |
| 1 | 136 (18%) | 20 (13%) |
| 2 | 54 (7%) | 11 (7%) |
| 3 | 96 (13%) | 15 (9%) |
| 4 | 63 (8%) | 14 (9%) |
| 5 | 75 (10%) | 12 (8%) |
| 6 | 246 (33%) | 55 (35%) |
| 7 | 21 (3%) | 3 (2%) |
| 8 | 53 (7%) | 27 (17%) |

**Supplementary Table 4: Cox-regression analysis on patients aged ≥80 years for overall survival**

| **Baseline Characteristics** | | ***HR** | **HR (CI)** | | **p-value** |
| --- | --- | --- | --- | --- | --- |
| **Route to diagnosis** | GP referral | Ref | - | - | - |
|  | Emergency presentation | **1.82** | **1.40** | **2.36** | **<0.01** |
|  | Inpatient elective | 0.41 | 0.10 | 1.68 | 0.22 |
|  | Other outpatient | 88 | 0.58 | 1.34 | 0.55 |
|  | NHS urgent cancer referral pathway | 0.93 | 0.72 | 1.20 | 0.60 |
|  | Unknown | 0.95 | 0.30 | 3.03 | 0.94 |
|  |  |  |  |  |  |
| **Age** | Age band | **1.06** | **1.02** | **1.11** | **<0.01** |
|  |  |  |  |  |  |
| **Sex** | Male | Ref | - | - | - |
|  | Female | **0.82** | **0.67** | **0.99** | **0.04** |
|  |  |  |  |  |  |
| **Year (2013-2015)** | Yearly increment | 0.96 | 0.85 | 1.09 | 0.52 |
|  |  |  |  |  |  |
| **Deprivation quintile** | Increment of quintile | **1.05** | **0.98** | **1.12** | **0.15** |
|  |  |  |  |  |  |
| **Stage** | Early | Ref | - | - | - |
|  | Late | **1.31** | **1.07** | **1.62** | **0.01** |
|  | Unknown | **1.24** | **0.92** | **1.67** | **0.16** |
|  |  |  |  |  |  |
| **Comorbidity index** | Increment of index | 1.05 | 0.97 | 1.15 | 0.24 |
|  |  |  |  |  |  |
| **Regimen** | R-CHOP | Ref | - | - | - |
|  | R-miniCHOP | 0.95 | 0.73 | 1.22 | 0.68 |

**Supplementary Figure 1: Flow diagram Data extraction and analysis**

**Supplementary Figure 2: Overall survival according to both stage and route of DLBCL presentation**

**Supplementary Figure 3A: OS by Diagnosis to treatment interval (DTI)**

**Supplementary Figure 3B: Diagnosis to treatment by route of referral**

**Supplementary Figure 4: Lymphoma-Specific Survival for R-CHOP and R-miniCHOP in patients ≥80 years**

**Supplementary Figure 5: Overall survival non-anthracycline treated patients by: a) all patients b) by age 65-79, ≥80** **years c) Route to Diagnosis**

b

a

c

**Supplementary Figure 6: Overall survival for all R-CVP and R-CEOP treated patients**

b

a

c
